# Improving the Thermoelectric Properties of the Half-Heusler Compound VCoSb by Vanadium Vacancy

**DOI:** 10.3390/ma12101637

**Published:** 2019-05-20

**Authors:** Lihong Huang, Junchen Wang, Xiaobo Mo, Xiaobo Lei, Sude Ma, Chao Wang, Qinyong Zhang

**Affiliations:** 1 Key Laboratory of Fluid and Power Machinery of Ministry of Education, School of Materials Science & Engineering, Xihua University, Chengdu 610039, China; huang.lihong@foxmail.com (L.H.); wangjunchen94@163.com (J.W.); moxiaobo77@163.com (X.M.); leixiaoboxhu@163.com (X.L.); masude2007@163.com (S.M.); 2 Clean Energy Materials and Engineering Center, School of Electronic Science and Engineering, State Key Laboratory of Electronic Thin Film and Integrated Devices, University of Electronic Science and Technology of China, Chengdu 610054, China

**Keywords:** Half-Heusler, VCoSb, vacancy, nonstoichiometry, thermoelectric

## Abstract

The effects of V vacancy on the thermoelectric performance of the half-Heusler compound VCoSb have been investigated in this study. A certain amount of CoSb secondary phase is generated in the VCoSb matrix when the content of V vacancy is more than 0.1 at%. According to the results, a *ZT* value of 0.6, together with a power factor of 29 μW cm^−1^ K^−2^ at 873 K, were achieved for the nonstoichiometric sample V_0.9_CoSb. This proved that moderate V vacancy could improve the thermoelectric (TE) properties of VCoSb. The noticeable improvements are mainly owing to the incremental Seebeck coefficient, which may benefit from the optimized carrier concentration. However, too much V vacancy will result in more CoSb impurity and deteriorate the TE performances of VCoSb owing to the increased thermal conductivity.

## 1. Introduction

Thermoelectric (TE) materials are a type of functional material that can be used to directly convert thermal energy into electrical energy through the motion of internal carriers in solids. The conversion efficiency of TE materials is characterized by the dimensionless figure of merit *ZT* = *S*^2^*σT*/(*κ*_e_ + *κ_L_*), where *S* is the Seebeck coefficient, *σ* is the electrical conductivity, *T* is the absolute temperature, and *κ*_e_ and *κ_L_* are the electronic and lattice contributions to the total thermal conductivity *κ*, respectively [1]. Particularly, the above three TE parameters (*S*, *σ*, and *κ*) are correlated with each other by carrier concentration. An effective way to enhance *ZT* is by optimizing carrier concentration.

According to the definition of *ZT*, there are two strategies for *ZT* enhancement: One strategy is increasing the TE power factor (*S*^2^*σ*) via band engineering, for example, modifying electron states by resonant levels [2], energy band convergency [3], low band effective mass [4], or weakening the carrier scattering [5]. The other strategy is to reduce *κ_L_*, e.g., alloying to induce point defects [6], increasing phonon scattering with nanostructure [7], or using materials with strong anharmonicity [8] and low sound velocity [9].

Over the last few decades, half-Heusler (HH) compounds, a type of thermoelectric material, have been given a lot of attention for their desirable characteristics, such as excellent mechanical robustness, good TE performance, high-temperature stability, and low toxicity, all of which are of great significance for TE materials’ practical applications [10,11,12]. The properties of HH compounds are strongly influenced by the number of valence electrons in a unit cell; HH compounds with a number of 18 valence electrons are typically semiconductors, and M_I_NiSn, M_I_CoSb (M_I_ = Ti, Zr, Hf), M_II_FeSb (M_II_ = Nb, V), and their alloys have been extensively investigated as promising TE materials applicated at medium-high temperature [13,14,15,16,17,18,19,20,21,22].

Unconventionally, experiments have confirmed that NbCoSb and VCoSb, with a cubic HH structure and 19 valence electrons per unit cell, exhibit moderate n-type TE properties and achieve a *ZT* value of about 0.4 and 0.5 at 973 K [23,24]. However, impurity phases always exist in a NbCoSb system and obstruct the improvement of its TE properties [25,26,27]. Moreover, Zeier et al. presented that NbCoSb with stoichiometric composition is unstable, and the nonstoichiometric HH compound Nb_0.8_CoSb with 18-electron and Nb vacancies was pointed out to be a more stable semiconductor [28]. More recently, Zhu et al. confirmed experimentally that Nb_0.8_CoSb is a stable HH compound, and the carrier concentration can be improved by the content of Nb vacancy. A peak *ZT* of about 0.9 at 1123 K was achieved for Nb_0.83_CoSb [29].

In this work, we focus on another HH compound, the thermoelectric material VCoSb with nominal VEC = 19 (VEC is the valence electron count per unit cell). Inspired by previous research on NbCoSb, we have synthesized V_1−*x*_CoSb compounds with different contents of V vacancies (*x* = 0, 0.05, 0.1, 0.15, 0.2, and 0.25), and investigated the effects of V vacancy on the TE properties. According to our experiment results, too much V vacancy will generate more impurity phase (CoSb), leading to a reduction in the TE performance. It is noteworthy that a certain amount of V vacancy in VCoSb will enhance the power factor and increase *ZT* of VCoSb. A peak *ZT* value of about 0.6 was achieved for V_0.9_CoSb at 973 K. The present work again demonstrates that nonstoichiometric 19-valence electron HH compounds are hopeful TE materials.

## 2. Experimental Details

A series of V_1−*x*_CoSb samples with different contents of V vacancies (*x* = 0, 0.05, 0.1, 0.15, 0.2, and 0.25) were synthesized by powder sintering, ball milling, and hot pressing. The raw powder materials were loaded into a stainless-steel jar, mixed for 0.5 h without grinding balls, and then consolidated into disks by cold pressing at room temperature for 20 min. The pre-compression disks were sealed in a quartz tube, sintered at 1073 K for 18 h, and then air cooled to room temperature. The sintered disks were sealed inside a jar in a glove box with argon gas protection, and then ball milled for 5 h by a high-energy SPEX 8000M Mixer/Mill (SPEX Sample Prep., Metuchen, NJ, USA) to yield the powder. The obtained powder was transferred and loaded into a graphite die, then hot-pressed at 1023 K for 2 min under 75 MPa by a direct current hot press to obtain a disk sample. The dense samples obtained were about 12.7 mm in diameter and 2 mm in thickness.

The thermal conductivity *κ* = *dDC*_p_ was calculated using the measured density (*d*) using the Archimedes method, thermal diffusivity (*D*) using the laser flash method (LFA 457, Netzsch, Selb, Germany), and specific heat capacity (*C*p) using differential scanning calorimetry (DSC 404 C, Netzsch, Selb, Germany). Bar-shaped samples of about 2 mm × 2 mm × 11 mm were used for measuring the electrical conductivity and Seebeck coefficient simultaneously from 300 to 973 K (ZEM-3, ULVAC Riko, Chigasaki, Japan).

The phase characterizations were measured by X-ray diffraction (XRD, D2 PHASER, Bruker, Billerica, MA, USA) with Cu K_α_ radiation. The microstructures were observed using a field emission scanning electron microscope (FESEM, QUANTA 250, FEI, Hillsboro, OR, USA).

## 3. Results and Discussion

**Figure 1:**
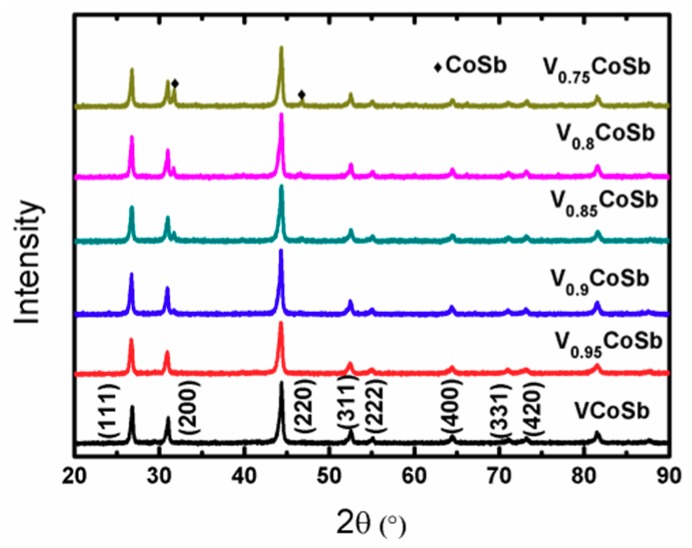
X-ray diffraction (XRD) patterns of V_1−*x*_CoSb samples.

Figure 1 shows the XRD patterns of the V_1−*x*_CoSb (*x* = 0–0.25) samples. As we can see, the main phase is the HH phase of the VCoSb with a cubic MgAgAs-type crystal structure. Although extremely weak impurity peaks of CoSb phase can be found in the V_0.9_CoSb sample, the stoichiometric VCoSb sample and the nonstoichiometric V_0.95_CoSb sample were found to be pure in phase. Obvious peaks of impurity phase of CoSb are observed when the content of V vacancy is more than 0.1, and the amount of the impurity phase of CoSb increases with the increasing content of V vacancy. Although there is a certain amount of V vacancies in our samples, the cubic HH phase crystal structure still remains. 

The lattice parameter, and the theoretical, experimental, and relative densities of the V_1−*x*_CoSb samples are shown in Table 1. The lattice parameter changes only slightly upon the increasing V vacancies, indicating no significant change in the main phase structure; which is in agreement with the results of the XRD patterns. The theoretical density is calculated by *d*_cal_ = ∑*n*_i_M_i_/(*a*^3^*N_A_*), where *n*_i_ is the number of atoms of V, Co, and Sb per unit cell, M_i_ is the corresponding atomic mass of each element, *a* is the lattice constant, and *N_A_* is the Avogadro constant (6.023 × 10^23^ mol^−1^). The relative densities of all of the hot-pressed samples were more than 93%.

**Figure 2:**
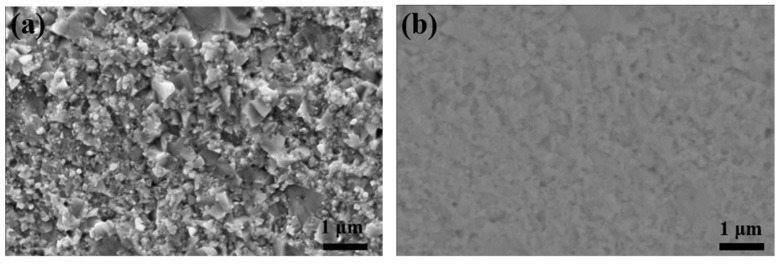
Scanning electron microscope (SEM) image (**a**) and back-scattering electron (BSE) image (**b**) of the hot-pressed sample V_0.9_CoSb.

As shown in Figure 2a, the scanning electron microscope (SEM) image of the fracture surface of V_0.9_CoSb sample indicates dense and compact hot-pressed bulk samples, and the grainsize of the sample is in a range from hundreds of nanometers to one micrometer. The back-scattering electron (BSE) image of V_0.9_CoSb in Figure 2b shows no apparent phase segregation, meaning very little secondary phase in the V_0.9_CoSb sample.

**Figure 3:**
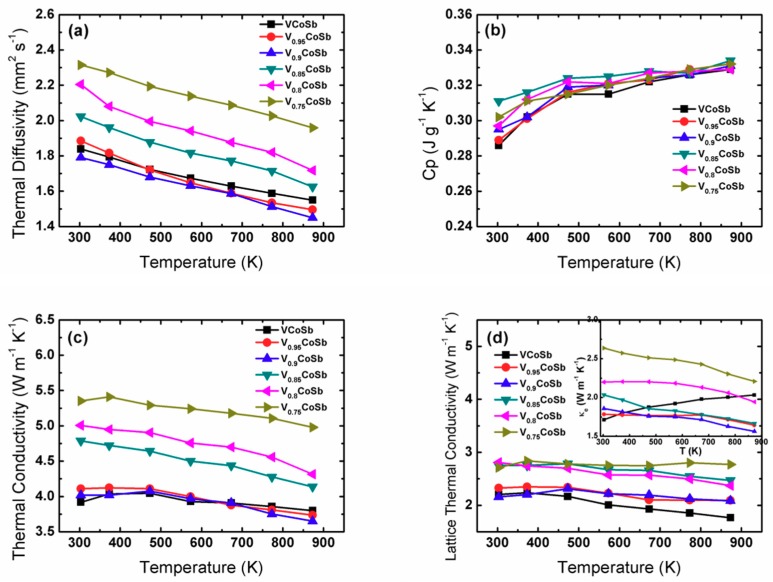
Temperature-dependent thermal diffusivity (**a**), thermal capacity (**b**), total thermal conductivity (**c**), and lattice and electronic thermal conductivity (**d**) of V_1−*x*_CoSb.

The temperature-dependent thermal properties are shown in Figure 3. Due to the limitation of our thermal performance tester system, the maximum test temperature was 873 K, which is lower than the maximum electrical test temperature of 973 K. As shown in Figure 3a, the thermal diffusivity *D* monotonically decreased over the whole measured temperature range. Additionally, the thermal diffusivity clearly increased when the V vacancy content is more than 0.15, which may be due to the increasing amount of CoSb secondary phase in the sample. As shown in Figure 3b, the larger the amount of CoSb, the higher the thermal capacity *C_p_*. Figure 3c shows the total thermal conductivity *κ*_total_ for V_1−*x*_CoSb samples as the temperature changes. *κ*_total_ of V_0.9_CoSb decreased with increasing temperature from 3.84 W m^−1^ K^−1^ at 300 K to 3.58 W m^−1^ K^−1^ at 873 K, almost close to that of VCoSb. Typically, the total thermal conductivity contains two parts, the electronic thermal conductivity and the lattice thermal conductivity, namely, *κ*_total_ = *κ_e_* + *κ_L_*. The electronic thermal conductivity *κ_e_* can be estimated by the Wiedemann–Franz relation, *κ_e_ = LσT*, where *L* is the Lorentz number. Usually, the Lorentz number is between two limiting values. For metals and highly degenerate semiconductors, it is 2.45 × 10^−8^ Ω W K^−2^, whereas, for a non-degenerate semiconductor, it is 1.49 × 10^−8^ Ω W K^−2^. Herein, the Lorentz number is estimated by fitting the measured Seebeck coefficient using a single parabolic band (SPB) model, as shown in Figure S1, assuming the acoustic phonon scattering mechanism and Equations (1)–(3) are valid, instead of using a constant value of 2.45 × 10^−8^ Ω W K^−2^ [30].

(1) $$S=\pm \frac{k_{B}}{e}\left(\frac{\left(r+5/2\right)F_{r+3/2}\left(\eta \right)}{\left(r+3/2\right)F_{r+1/2}\left(\eta \right)}-\eta \right)$$

(2) $$F_{n}\left(\eta \right)=\int_{0}^{\infty }\frac{x^{n}}{1+e^{x-\eta }}dx$$

(3) $$L={\left(\frac{k_{B}}{e}\right)}^{2}\left[\frac{\left(r+7/2\right)F_{r+5/2}\left(\eta \right)}{\left(r+3/2\right)F_{r+1/2}\left(\eta \right)}-{\left(\frac{\left(r+5/2\right)F_{r+3/2}\left(\eta \right)}{\left(r+3/2\right)F_{r+1/2}\left(\eta \right)}\right)}^{2}\right]$$

where *F_n_*(*η*) is the *n*th order Fermi integral, *η* is the reduced Fermi energy, *r* is the scattering factor, *h* is Plank’s constant, *k*_B_ is Boltzmann’s constant, *x* is the variable of integration, and *e* is the electron charge.

The obtained *κ_L_* and *κ_e_* are plotted in Figure 3d. *κ_L_* exhibits significant enhancement with increasing V vacancy, i.e., increasing amounts of CoSb impurity. For example, *κ_L_* at room temperature increased from 2.32 W m^−1^ K^−1^ for the V_0.95_CoSb sample to 3.41 W m^−1^ K^−1^ for the V_0.8_CoSb sample. As shown in the inset of Figure 3d, *κ_e_* of VCoSb showed an increasing trend over the whole temperature range; however, it decreased with the increasing temperature for V-deficient samples V_1−*x*_CoSb (*x* > 0). Furthermore, *κ_e_* at room temperature distinctly increased from 1.75 W m^−1^ K^−1^ for VCoSb to 2.64 W m^−1^ K^−1^ for V_0.75_CoSb upon the increasing V vacancy content, which can be ascribed to the high electrical conductivity of the CoSb impurity.

**Figure 4:**
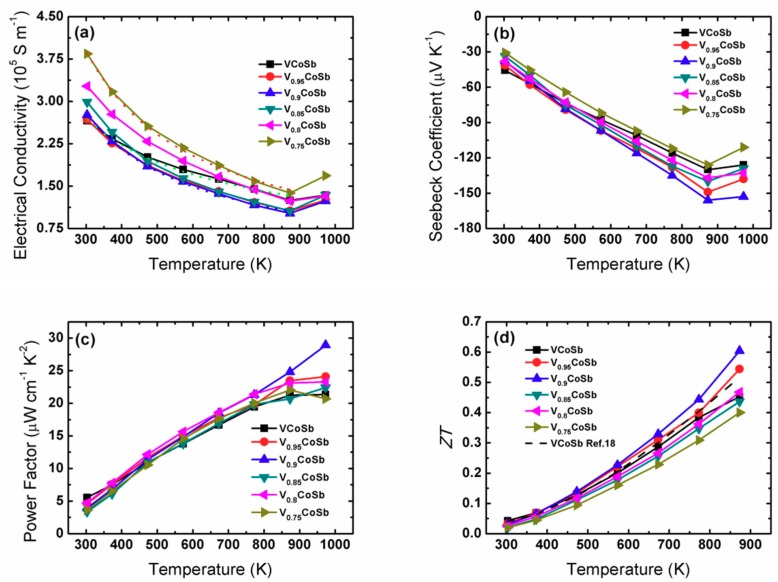
Temperature-dependent electrical conductivity (**a**), Seebeck coefficient (**b**), power factor (**c**), and *ZT* (**d**) of V_1−*x*_CoSb.

Figure 4a shows the temperature dependence of electrical conductivity of the V_1−*x*_CoSb samples. The electrical conductivity decreased at the beginning with the increasing temperature due to the strong electron scattering, displaying metal or degenerate semiconductor transport behavior. However, after 873 K the electrical conductivity started to increase, which may be due to the intrinsic carrier thermal excitation. We calculated the temperature-dependence-exponent of the conductivity (*σ* vs. *T^α^*) before thermal excitation and found that the carrier scattering mechanism may change after inducing V vacancy. The conductivity of VCoSb showed a temperature exponent of −0.65 (green dotted line), indicative of alloy and phonon scattering of the carriers, although alloy scattering is stronger. However, the temperature exponent of −0.9 for V_0.9_CoSb (blue dotted line) and −0.93 for V_0.75_CoSb (red dotted line) implies that phonon scattering has a stronger effect (*σ* vs. *T*^−0.5^ for alloying scattering and *σ* vs. *T*^−1.5^ for acoustic phonon scattering) [31,32].

We also prepared pure phase CoSb, and characterized its electrical properties, as shown in Figure S2. The bulk CoSb sample had a density of 8.63 g/cm^3^, and a room temperature conductivity of 37.5 × 10^5^ S/m. Based on its conductivity dependence on temperature (*σ* vs. *T*^−1.5^), which implies that acoustic phonon scattering is predominant during the carrier transport, the carrier concentration here is considered to be nearly temperature-irrelevant due to the metallic nature of carriers. Therefore, too much CoSb impurity in the matrix will degrade the TE performance of VCoSb, compared with other secondary phases, such as ZnO and SiC [33,34]. CoSb is not a suitable scattering phase because of its strong metallic properties.

As shown in Figure 4b, the negative Seebeck coefficients indicate an n-type transport behavior of our samples. Moreover, the Seebeck coefficient enhanced with the increase of temperature before 873 K and then decreases on account of intrinsic thermal excitation. The Seebeck coefficient for the V vacancy samples is larger than that of VCoSb, except for V_0.75_CoSb. The V_0.9_CoSb sample has the highest peak Seebeck coefficient of –153 μV/K at 873 K, which should benefit from the improved carrier concentration.

Consequently, the power factor (*S*^2^*σ*) increased during the whole temperature range and the V_0.9_CoSb sample had an improved peak value of 29 μW cm^−1^ K^−2^; greater than VCoSb (21 μW cm^−1^ K^−2^) at 973 K (Figure 4c). Finally, the *ZT* values are presented in Figure 4d. It is apparent that the V_0.9_CoSb sample showed the highest *ZT* of 0.6 at 873 K, nearly a 25% enhancement compared to VCoSb, on account of the improved Seebeck coefficient and power factor.

## 4. Conclusion

A set of V vacancy V_1−*x*_CoSb samples (*x* = 0, 0.05, 0.1, 0.15, 0.2, and 0.25) were prepared by powder sintering, ball milling, and hot pressing. The influences of V vacancy on the thermoelectric properties of the HH compound VCoSb were studied. A certain amount of CoSb secondary phase was generated in the VCoSb matrix when x was more than 0.1. Too much V vacancy yields more CoSb impurity, and deteriorated the TE performance, owing to the increased thermal conductivity; while moderate V vacancy could improve the TE property, which may benefit from the optimized carrier concentration. Finally, a maximum *ZT* of about 0.6 was obtained at 873 K for V_0.9_CoSb, mainly because of the increased Seebeck coefficient compared with VCoSb.

## Figures and Tables

**Table 1:** Lattice parameter and theoretical, experimental, and relative density of V_1−*x*_CoSb (*x* = 0, 0.05, 0.1, 0.15, 0.2, and 0.25) samples.

Nominal Composition	Lattice Parameter (nm)	Density (g/cm^3^)		Relative Density (%)
		Theoretical	Experimental	
VCoSb	0.5772	7.45	8.01	93.01
V_0.95_CoSb	0.5772	7.53	7.87	95.67
V_0.9_CoSb	0.5771	7.6	7.79	97.56
V_0.85_CoSb	0.5770	7.62	7.73	98.57
V_0.8_CoSb	0.5770	7.63	7.68	99.35
V_0.75_CoSb	0.5769	7.64	7.66	99.74

## Supplementary Materials

The following are available online at https://www.mdpi.com/1996-1944/12/10/1637/s1, Figure S1: The Lorentz number estimated by fitting the Seebeck coefficient, Figure S2: Temperature-dependent electrical conductivity (**a**), Seebeck coefficient (**b**), power factor (**c**), and XRD pattern (**d**) of CoSb.
