# Supplementary material for: Improving the Thermoelectric Properties of the Half-Heusler Compound VCoSb by Vanadium Vacancy
Source: Materials (Basel). 2019 May 20;12(10):1637. doi: 10.3390/ma12101637 (PMC6566434; doi:10.3390/ma12101637)
Supplement: Supplementary file 1 [file materials-12-01637-s001.pdf]

Supplementary Materials: Improving the Thermoelectric Properties of the Half-Heusler Compound VCoSb by Vanadium Vacancy

Lihong Huang [1,2], Junchen Wang [1], Xiaobo Mo [1], Xiaobo Lei [1], Sude Ma [1], Chao Wang [2,*], and Qinyong Zhang [1,*]

[1] Key Laboratory of Fluid and Power Machinery of Ministry of Education, School of Materials Science & Engineering, Xihua University, Chengdu 610039, China; huang.lihong@foxmail.com (L.H.); wangjunchen94@163.com (J.W.); moxiaobo77@163.com (X.M.); leixiaoboxhu@163.com (X.L.); masude2007@163.com (S.M.)

[2] Clean Energy Materials and Engineering Center, School of Electronic Science and Engineering, State Key Laboratory of Electronic Thin Film and Integrated Devices, University of Electronic Science and Technology of China, Chengdu 610054, China

* Correspondence: cwang@uestc.edu.cn (C.W.); bohr123@163.com (Q.Z.)

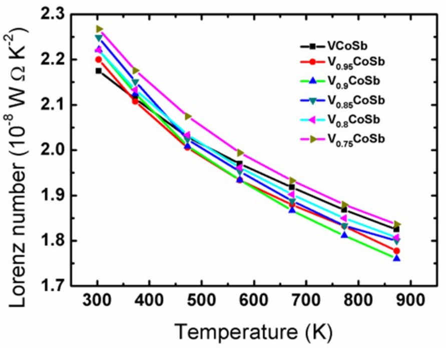

Figure S1. The Lorentz number estimated by fitting the Seebeck coefficient.

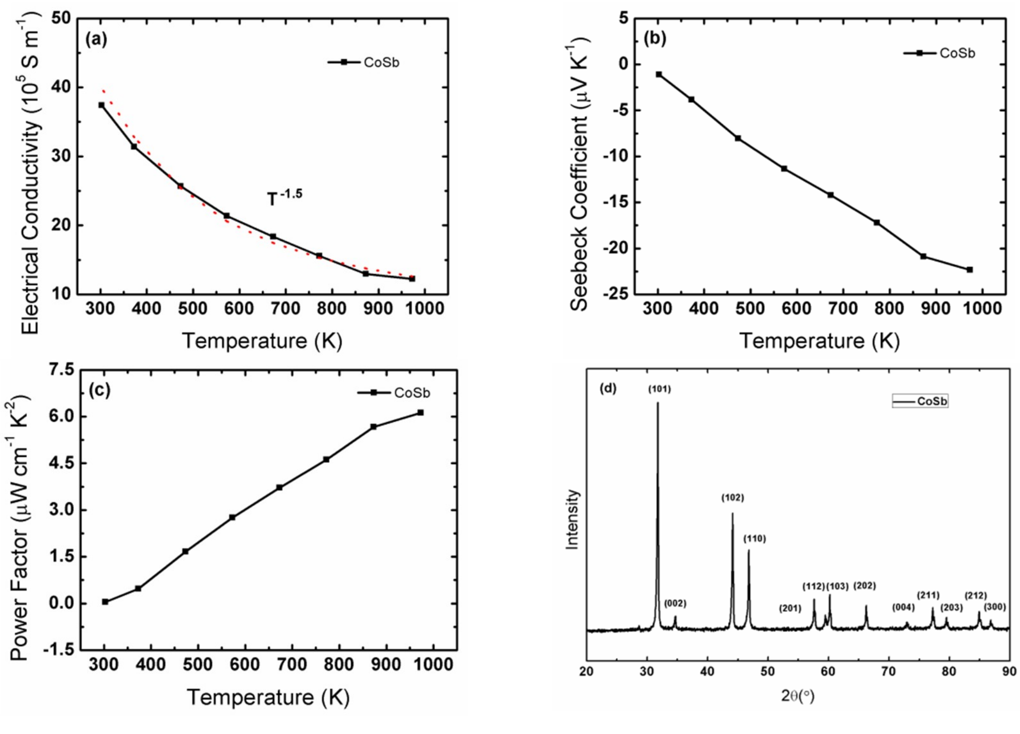

Figure S2. Temperature-dependent electrical conductivity (**a**), Seebeck coefficient (**b**), power factor (**c**), and XRD pattern (**d**) of CoSb.
